# Miscellany and Literary Notes

**Published:** 1895-04

**Authors:** 


THE *American Lancet*, in its March issue, 1895, announces its discontinuance. Dr. Leartus Connor, who has been conducting editorial work for the past twenty-four years, much of the time as editor of the *Lancet*, has resigned, hence the publisher, Mr. Geo. S. Davis, suspends its publication.

There are too many medical journals, to be sure, but there are many that we could afford to give up before the *American Lancet.* It was a dignified journal, conducted by an able editor and we shall be sorry to miss it from our exchange list.

---

MESSRS. SCHULZE-BERGE & KOECHL, 79 Murray street, New York, announce that on and after April 1st they will again reduce the price of diphtheria antitoxin Behring, as follows:

| | | | | |
|---|---|---|---|---|
| No. 0, yellow label, 200 antitoxin units | | | ................ | $ .60 |
| No. 1, green label, 600 | " | " | ................ | 1.50 |
| No. 2, white label, 1,000 | " | " | ................ | 2.75 |
| No. 3, red label, 1,500 | " | " | ................ | 3.75 |

Supplied direct upon receipt of price.

---

THE oldest medical publications in the United States, in the order named, are thus given by the *New Orleans Medical and Surgical Journal:* The *Boston Medical and Surgical Journal*, the *American Journal of Medical Sciences*, the *Cincinnati Lancet-Clinic*, the *St. Louis Medical and Surgical Journal* and the *New Orleans Medical and Surgical Journal.*

The first number of the BUFFALO MEDICAL JOURNAL was published in 1845, under the editorship of Dr. Austin Flint. This JOURNAL will, therefore, be fifty years old in June. We presume, if the computation of our New Orleans contemporary is correct, we are entitled to the sixth place in chronological order.

MESSRS. WM. R. WARNER & Co. have recently issued a therapeutic ready reference book for physicians, seventh edition, which is a valuable aid in the preparation of prescriptions and gives many useful hints on the subject of prescription writing. It will be sent to any subscriber of the JOURNAL who will remit 15 cents to the publishers, 1228 Market street, Philadelphia.

---

THE Medical Novelty Co., 21 West Twenty-third street, New York, has sent us a copy of their physicians' pocket day-book and visiting list. This book is both convenient and useful for every physician. There is a large demand for them and they will be supplied free throughout the year to any one of our subscribers who will send their names to the above address, mentioning the JOURNAL.

---

THE *Southern California Practitioner* appeared in January, 1895, in a new dress, which gives it an improved appearance. Its pages are full of interesting reading matter.

---

ANNALES D'OCULISTIQUE.—It will be gratifying to the profession, particularly the ophthalmological branch of it, to learn that the *Annales d' Oculistique*, the oldest and one of the best ophthalmic periodicals in existence, is hereafter to be published simultaneously and uniformly each month in the French and English languages. This plan of publication began with January, 1895, and the English edition is brought out at $5 a year. Each number contains sixty-four to eighty pages, forming annually two volumes of about 1,000 pages.

This periodical was founded in 1838 by Cunier, and was a pioneer in this department of medical journalism. From the beginning its fame and influence has extended, and through the wise direction of its founder, and later of Warlomont and now of Valude, it has been a leader in ophthalmological thought and has held a foremost rank in all countries where ophthalmology has been studied. It is distinguished for its dignity, independence and enterprise and in offering a complete record of this branch of medical science. It is the wish of the management to make the journal in every respect an international medium of communication between ophthalmic practitioners. In future issues original communications from American and English writers will constitute a much more prominent feature than in the past.

This new enterprise will be most welcome and it will, without any doubt, receive that generous support which it deserves. H.

